# Biophysical characterization data on Aβ soluble oligomers produced through a method enabling prolonged oligomer stability and biological buffer conditions

**DOI:** 10.1016/j.dib.2015.07.034

**Published:** 2015-08-06

**Authors:** Amanda C. Crisostomo, Loan Dang, Jyothi L. Digambaranath, Andrea C. Klaver, David A. Loeffler, Jeremiah J. Payne, Lynnae M. Smith, Adam L. Yokom, John M. Finke

**Affiliations:** a Sciences and Mathematics, IAS, University of Washington, Tacoma, WA 98402, United States; b Eye Research Institute, Oakland University, Rochester, MI 48309, United States; c Department of Chemistry, Oakland University, Rochester MI 48309, United States; d Department of Neurology Research, Beaumont Health System, Royal Oak MI, United States

## Abstract

The data here consists of time-dependent experimental parameters from chemical and biophysical methods used to characterize Aβ monomeric reactants as well as soluble oligomer and amyloid fibril products from a slow (3–4 week) assembly reaction under biologically-relevant solvent conditions. The data of this reaction are both of a qualitative and quantitative nature, including gel images from chemical cross-linking and Western blots, fractional solubility, thioflavin T binding, size exclusion chromatograms, transmission electron microscopy images, circular dichroism spectra, and fluorescence resonance energy transfer efficiencies of donor–acceptor pair labels in the Aβ chain. This data enables future efforts to produce the initial monomer and eventual soluble oligomer and amyloid fibril states by providing reference benchmarks of these states pertaining to physical properties (solubility), ligand-binding (thioflavin T binding), mesoscopic structure (electron microscopy, size exclusion chromatography, cross-linking products, SDS and native gels) and molecular structure (circular dichroism, FRET donor-acceptor distance).

Aβ1-40 soluble oligomers are produced that are suitable for biophysical studies requiring sufficient transient stability to exist in their “native” conformation in biological phosphate-saline buffers for extended periods of time. The production involves an initial preparation of highly monomeric Aβ in a phosphate saline buffer that transitions to fibrils and oligomers through time incubation alone, without added detergents or non-aqueous chemicals. This criteria ensures that the only difference between initial monomeric Aβ reactant and subsequent Aβ oligomer products is their degree of peptide assembly. A number of chemical and biophysical methods were used to characterize the monomeric reactants and soluble oligomer and amyloid fibril products, including chemical cross-linking, Western blots, fraction solubility, thioflvain T binding, size exclusion chromatography, transmission electron micrscopy, circular dichroism spectroscopy, and fluorescence resonance energy transfer.

Specifications Table

**Table:** 

Subject area	Biochemistry
More specific subject area	Analytical Biochemistry
Type of data	Text file and figures
How data was acquired	Size-exclusion chromatography (SEC): Sephacryl S-200HR column (GE Life Sciences) with a Pharmacia peristaltic pump and Gilson Fraction collector. Gel imaging: Odyssey Infrared Imaging System (LI-COR Biosciences, Lincoln, NE), Gel Logic 100 Digital Imaging System (Kodak) using a Dark Reader Transilluminator (Clare Chemical). Absorbance: Cary 100 spectrophotometer (Cary). Steady-state fluorescence: PTI Quanta Master Steady-State fluorimeter (Photon Techonologies Inc). Time-resolved fluorescence: PTI EasyLife equipped with a 295 nm LED excitation source and 395 cut-on emission filter. CD spectra: Chirascan spectropolarimeter (Applied Photophysics). Electron microscopy: Morgagni 268 Transmission Electron Microscope (TEM) equipped with a Hamamatsu digital camera.
Data format	Analyzed
Experimental factors	All samples were measured either directly or with preparation steps essential to the actual experiment. Specifically (1) PICUP experiments involved raw sample treatment with cross-linking agent Tris(2,2′-bipyridyl)dichlororuthenium(II); (2) Thioflavin T fluorescence was measured after raw sample dilution into 25 µM ThT; (3) Samples for electron microscopy are fixed with glutaraldehyde and dried on a formar grid.
Experimental features	Size exclusion chromatography
Data source location	Tacoma, Pierce County, Washington State, USA
Data accessibility	Data is with this article.

## 1 Value of the data

(1) The data provides descriptive experimental biophysical parameters obtained on a specific preparation method of Aβ monomers, soluble oligomers, and insoluble aggregates.
(2) The preparation method described is used to facilitate a slow Aβ assembly process under biologically-consistent solvent conditions that also enable extensive study of initial monomers during the first 2–3 days and a stable oligomer/aggregate equilibrium at longer times.
(3) The data provides a means by which future Aβ oligomer preparations can confirm a similar oligomer macroscopic and molecular structure as that used in the present article

## 2 Data, experimental design, materials and methods

The data of the present article provides a biochemical and biophysical parameters that characterize Aβ soluble oligomers produced through a specific protocol and also measured a various time points during the oligomer incubation. The data included are from experiments of Photo-Induced Cross-Linking of Proteins (PICUP), Fraction Soluble Protein, Thioflavin T (ThT) binding, Size Exclusion Chromatographic (SEC) data, Transmission Electron Microscopy (TEM) images, Western Blot images, Circular Dichroism (CD) spectroscopy spectra, and fluorescence resonance energy transfer (FRET) efficiencies. There experiments were performed to characterize the time stages during the monomer-to-oligomer transition and subsequent oligomer isolation from the final oligomer/fibril product mixture.

This data is important as a wide variation of Aβ soluble oligomer forms have been shown to result from different preparation conditions [1]. Thus, the value of the data is to facilitate future scientific efforts that require replication of this particular oligomer species. The data here pertain to a specific form of “native” oligomers (nOAβ) whose production follows the following criteria: (1) the nOAβ preparation initially starts with highly pure MAβ in>99% molar excess phosphate-saline buffer and (2) nOAβ must be generated from this initial MAβ preparation by time alone (no change in solution conditions or seeding). These criteria were selected to ensure that the only difference between preparations corresponding to the initial Aβ monomers (MAβ) and subsequently formed nOAβ oligomers is the degree of peptide assembly. Solvent conditions remain constant and oligomer formation is driven by inherent kinetic mechanisms from the monomer state without influence of amyloid “seeds”.

## 3 Data

**Fig. 1:**
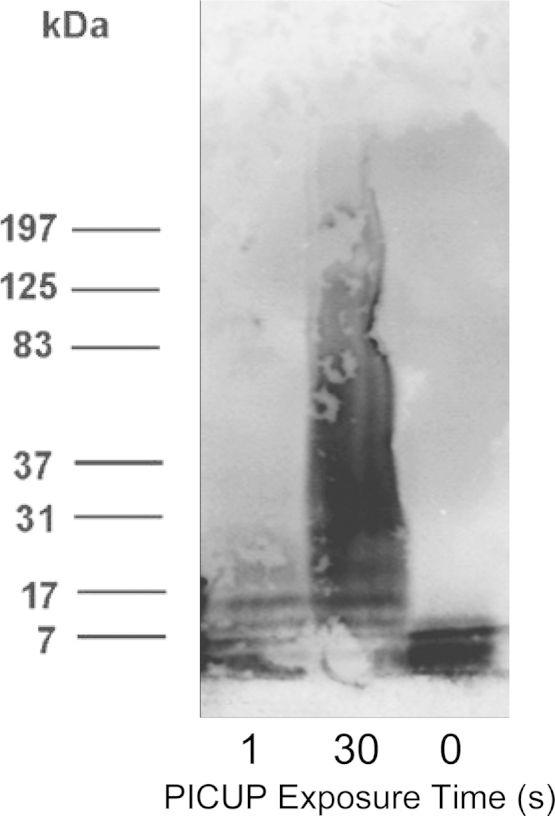
Aβ monomer preparations are of high purity. SDS-PAGE analysis of Photoinduced Cross-Linking of Unmodified Proteins (PICUP) treatment of freshly prepared monomeric Aβ (MAβ). The time of light exposure (in seconds) during PICUP is shown under each gel lane.

A highly pure initial MAβ preparation is a criteria for the production of nOAβ. Fig. 1 confirms this initial state with SDS-PAGE Western blots performed on PICUP experiments with *t*=0, 1, and 30 s of light exposure. The gel in Fig. 1 confirms the expected results of a single band at the expected monomer mass (4.3 kD) in the negative control samples with no light exposure (0 s lane) and a broad ladder of oligomer bands in the positive control (30 s lane). The integrated signal of oligomers in the 30 s lane was approximately equal to the 0 sec PICUP lane indicating that no larger insoluble aggregates had formed at exposure times less than 30 sec. Also, the oligomer distribution from infrared fluorescence is consistent with that of the 30 s exposure of Aβ_1–40_ in the original PICUP study [2]. The PICUP experiment here used 1 s of light exposure and retained monomer band as the highest intensity band but also produced 2–5 fainter higher-order bands that decreased in intensity at greater size. Compared with previous PICUP results, this finding is consistent with a monomeric Aβ sample because low amounts of 2–5mer bands are expected to result from diffusional collisions of monomeric polypeptides [2].

**Fig. 2:**
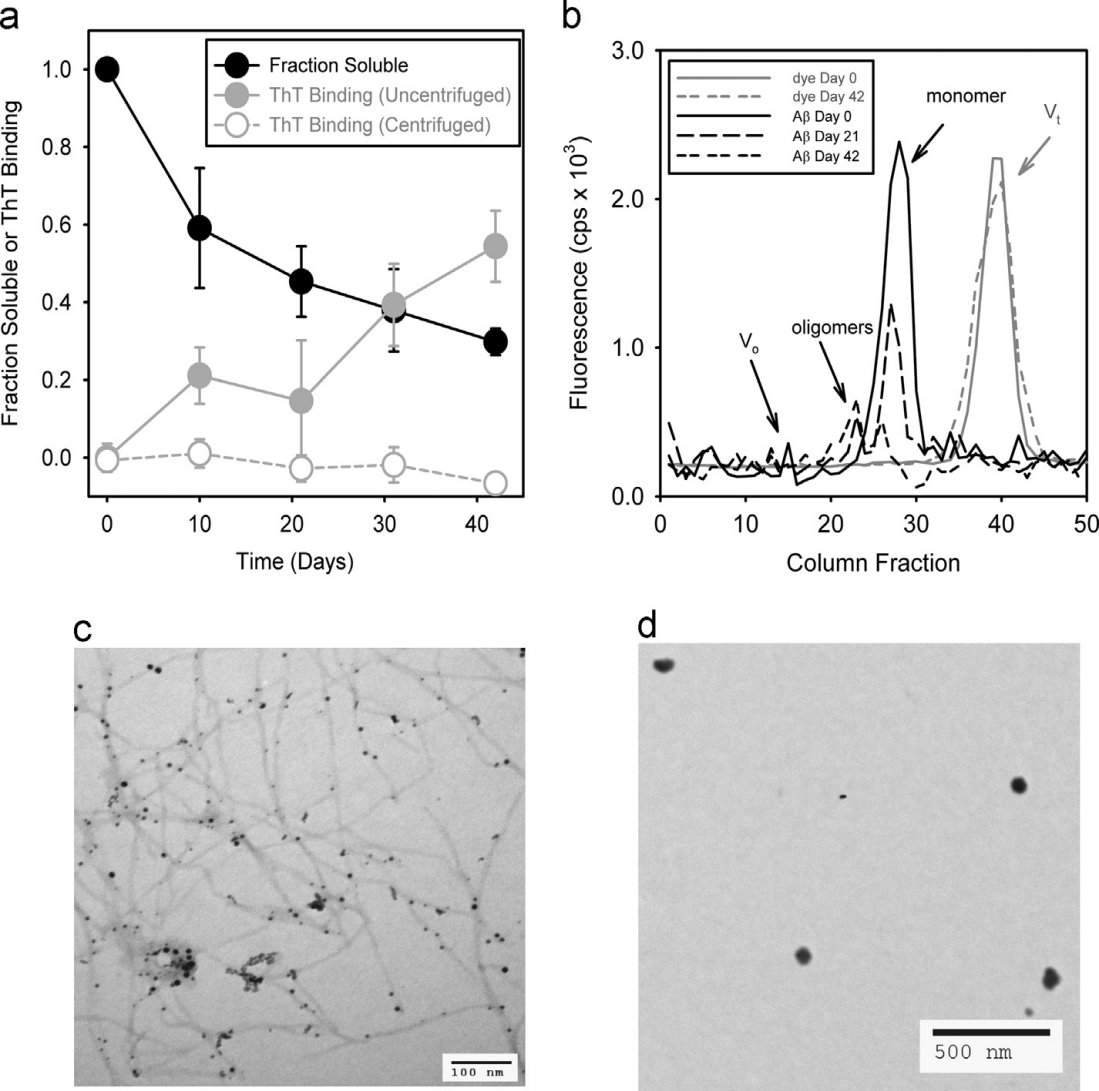
Aβ oligomer production and purification from an initial Aβ monomer preparation. (a) Fraction Soluble Aβ (▬●▬) and Thioflavin T binding () of the monomer-to-oligomer incubation every ten days. Thioflavin T binding was also measured for centrifuged aliquots of the oligomer incubation (). (b) Size exclusion chromatography of 1 ml aliquots from the monomer-to-oligomer incubation run on Day 0 (▬▬▬), Day 21 (----------), and on Day 42 ( ∙∙∙∙∙∙∙∙∙∙∙∙∙∙∙ ). Also shown are control SEC measurements of fluorescent 10 μM aminobenzamide doped into the monomer-to-oligomer incubation on Day 0 () and on Day 42 (). Arrows indicate the void volume *V*_o_, total volume *V*_t_, and elution peaks associated with monomeric MAβ and oligomeric nOAβ. (c) A transmission electron microscopy image of fibrils and oligomers after 42 days of incubation. (d) A transmission electron microscopy image of nOAβ purified by SEC.

Analyses of the transition of the initial MAβ preparation into nOAβ and fibrillar Aβ (FAβ) over time are shown in Fig. 2. Fig. 2a shows that the Aβ Fraction Soluble decreases to 0.3 after 40 days, indicating the increasing population of insoluble aggregates. This decrease in Fraction Soluble occurred along with a rise in ThT binding, indicating that insoluble Aβ can be attributed to ThT-binding FAβ. This was further confirmed by the absence of enhanced ThT fluorescence in centrifuged samples, demonstrating that ThT fluorescence could not be attributed to nOAβ.

Fig. 2b shows the results of SEC on the nOAβ incubation at 0, 21, and 42 days. On Day 0, the incubation is entirely MAβ (Kav~0.6, fraction 28) but transitions to two early-eluting nOAβ peaks by Day 42 (Kav~0.35/fraction 22 and Kav~0.5/fraction 25) [3]. The fraction of soluble Aβ recovery (0.21) from SEC integrated peak areas on Day 42 was comparable to the Fraction Soluble (0.3) in Fig. 2a. The largest peak in fraction 22, containing ~0.15 of initial Aβ, was collected as the final product for biophysical studies and TEM analysis. To test any change in column performance with injected MAβ and nOAβ solutions, 10 µM of *o*-aminobenzamide (ABZ) was added to the Aβ incubations on Day 0 (MAβ) and Day 42 (nOAβ) and injected on the SEC column. The ABZ elution volume was used to assess changes in *V*_t_ in these two preparations. While some peak broadening was observed on Day 42, no change in the elution volume was observed.

TEM of the raw Aβ incubation on Day 42 shows that a significant level of FAβ along with spherical structures on or near the fibrils (Fig. 2c). Similar spherical structures are also observed in TEM scans of the SEC purified nOAβ (Fig. 2d). These TEM studies indicate that nOAβ co-populates with FAβ but is of sufficient stability to enable purification by SEC.

**Fig. 3:**
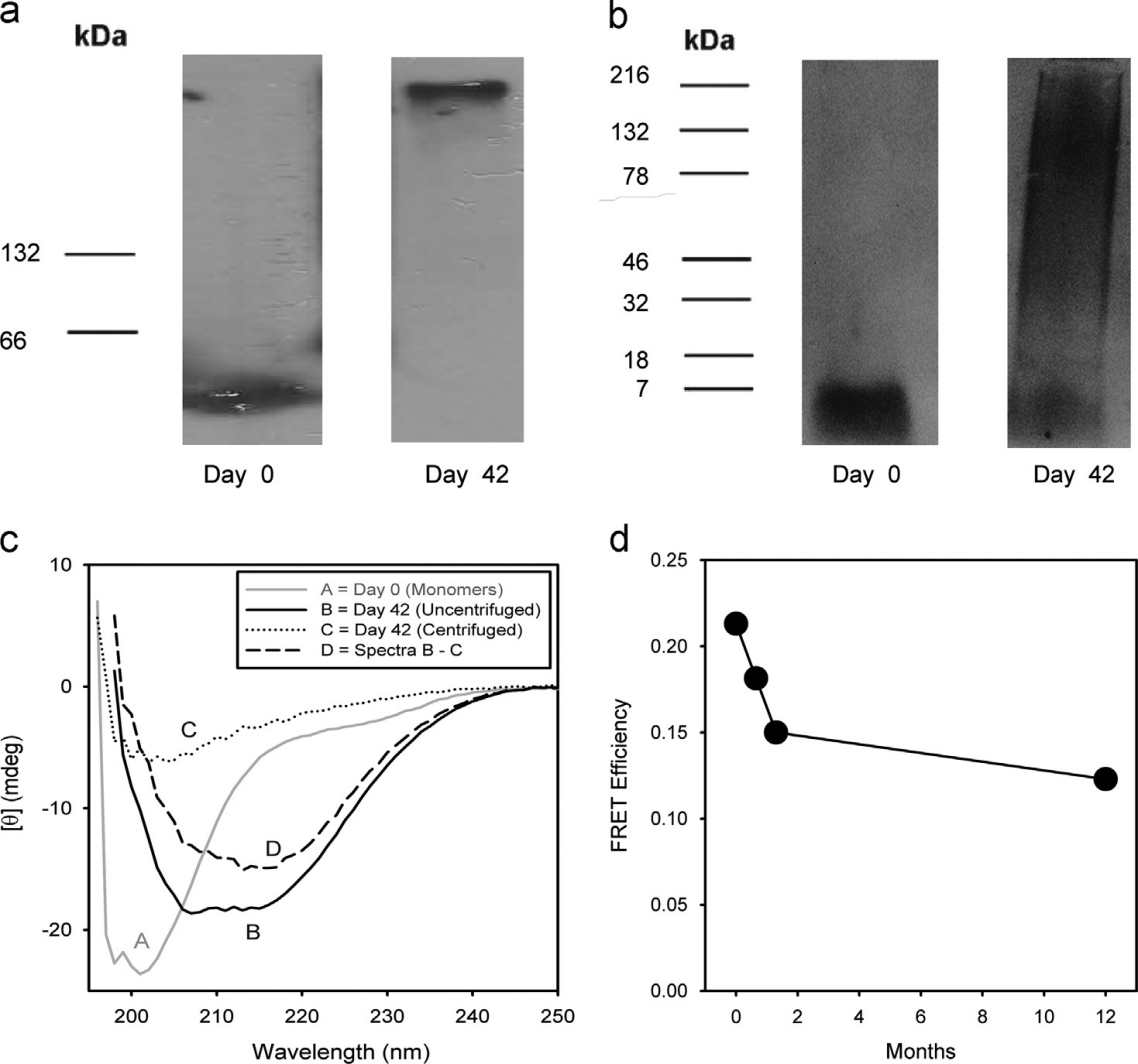
Structural analysis of oligomeric Aβ. (a) Native and (b) SDS-PAGE Western blots were imaged on Day 0 and on Day 42 of the Aβ monomer-to-oligomer incubation. (c) Uncorrected circular dichroism spectra were acquired on the Aβ monomer-to-oligomer incubation on Day 0 (A, ), Day 42 (B, ▬▬▬), and Day 42 after centrifugation (C, ∙∙∙∙∙∙∙∙∙∙∙∙∙∙∙). A subtracted spectra of B-C shows an estimated spectrum of insoluble Aβ on Day 42 (D, ----------). (d) FRET efficiency E between the aminobenzamide donor at residue 35 and nitrotyrosine acceptor at position 10 of FRET –labeled Aβ peptides in soluble states (MAβ and nOAβ) over the course of 365 days. The FRET-labeled Aβ was 2 μM (4%) of the total 50 μM Aβ sample, a dilution sufficient to observe only intramolecular FRET and no intermolecular FRET.

Analysis of the Aβ incubation on Day 0 by both native (Fig. 3a) and SDS-PAGE (Fig. 3b) Western Blots shows a single band consistent with the highly pure MAβ solution determined by PICUP (Fig. 1). On Day 42, native Western Blots show that the MAβ band is gone and replaced with a single large nOAβ band near the top of the gel (Fig. 3a). Under SDS-PAGE conditions, this nOAβ band partially denatures into sOAβ fragments (Fig. 3b).

A structural analysis of these Aβ states by CD (Fig. 3c) shows random coil MAβ conformations on Day 0 (gray spectrum A) with increased β-sheet structure on Day 42 when nOAβ and FAβ predominate (black spectrum B) [4]. Centrifugation of the Day 42 sample removes FAβ and other insoluble aggregates, leaving only nOAβ (dotted spectrum C). Subtraction of centrifuged from uncentrifuged Day 42 spectra reveals a spectra consistent with β-sheet conformations for FAβ (dashed spectrum D) [4]. Using the wavelength of minimum ellipticity (*λ*_minθ_) as a reaction coordinate for the structural transition between the MAβ coil (*λ*_minθ_ of *A*=201 nm) and the FAβ β-sheet (*λ*_minθ_ of *D*=216 nm), the nOAβ spectra (*λ*_minθ_ of *C*=203 nm) is more similar to MAβ.

An extended longitudinal analysis of nOAβ polypeptide conformational stability was performed using the FRET efficiency *E* between a fluorescent donor at position 35 and acceptor at position 10 (Fig. 3d). A rapid decrease in FRET efficiency occurs in the first 40 days of incubation as the soluble fraction transitions from MAβ (*E*=0.21) into nOAβ. (*E*=0.15). After soluble nOAβ has equilibrated with insoluble FAβ at 40 days, further incubation produces only minimal change after 11 months (*E*=0.12). While the results of Fig. 3d cannot rule out changes in the quarternary assembly states of nOAβ, the average molecular conformation of the constituent nOAβ peptides remains stable for at least one year.

## 4 Materials and methods

### 4.1 Instrumentation

Size-exclusion chromatography (SEC) was performed with a Sephacryl S-200HR column (GE Life Sciences) with a Pharmacia peristaltic pump and Gilson Fraction collector. Absorbance spectra were acquired on a Cary 100 spectrophotometer (Cary). Steady-state fluorescence measurements were acquired on a PTI Quanta Master Steady-State fluorimeter (Photon Techonologies Inc). Time-resolved fluorescence measurements were acquired on a PTI EasyLife equipped with a 295 nm LED excitation source and 395 cut-on emission filter. CD measurements were acquired on a Chirascan spectropolarimeter (Applied Photophysics). Electron microscopy was performed using a Morgagni 268 Transmission Electron Microscope (TEM) equipped with a Hamamatsu digital camera.

### 4.2 Monomeric and oligomeric Aβ preparation

All Aβ preparations involved initial production of monomeric Aβ (MAβ) with a slightly modified published protocol [5]. Commerical Aβ_1–40_ (Anaspec, San Jose) was dissolved in a 50:50 trifluoroacetic acid (TFA):hexafluoroisopropanol (HFIP) at a 1 mg/ml, sonicated for 1 h at room temperature, evaporated into a dry film under Argon gas, resuspended at 0.5 mg/ml peptide (114 μM) in aqueous TFA pH 3, microcentrifuged at 14,000×*g* for 10 min, and filtered through a 10,000 MWCO Amicon filter. The molar concentration of this MAβ stock solution was determined from UV spectroscopy using *ε*_280_=1280 M^−1^ cm^−1^ of Tyr10 [6]. For experiments, the MAβ stock was brought to 50 μM MAβ, 100 mM phosphate, 100 mM NaCl, 0.00075 M TFA, pH 7.4. For experimental analysis of MAβ or immobilization of MAβ for SPR, the MAβ stock was stored at 4 °C and used within 3 days of disaggregation.

To produce oligomeric Aβ (OAβ), the MAβ preparation was incubated at 25° C for a minimum of 40 days and this mature OAβ preparation used within 6 months. Prior to experimental analysis or SPR immobilization, OAβ was initially isolated from FAβ using 12,000×*g* centrifugation. OAβ in the supernatant was further purified using size-exclusion chromatography (SEC) calibrated with dextran blue (2000 kD), cytochrome c (13 kD), and sodium chromate (0.162 kD). SEC was conducted at 1 ml/min and eluting Aβ species were identified with intrinsic Tyr10 fluorescence (ex 280 nm, em 340 nm). The dominant OAβ peak was identified at approximately *K*_av_=0.35 and collected. OAβ purified in this manner was used for experiments or SPR immobilization within 1 week of elution from the column.

### 4.3 PICUP experiments

To confirm the absence of any minor OAβ and FAβ components in the initial preparation, Photo-Induced Cross-Linking of Unmodified Proteins (PICUP) was used [2]. A 40 μl solution of 1 μM ammonium persulfate, 50 μM Tris(2,2′-bipyridyl)dichlororuthenium(II), and 45 μM Aβ solution was prepared in the dark. The solution was then exposed to a 250 W tungsten lamp (GE) for 1 s in the chamber of a DS34 Polaroid Camera (F-stop set to 4.5). In the dark, crosslinking in the sample was quenched with the addition of 10 uL of 100 mM Tricine with 5% BME. A negative control was performed with no light exposure and positive control was performed with 30 s of light exposure. Samples were run on SDS-PAGE Western Blots.

### 4.4 Western blots

Both SDS and native gels used 4–20% Tris–HCl Ready Gels (Bio-Rad). For SDS-PAGE gels, Aβ samples were mixed 1:1 with Laemmli Sample Buffer (Bio-Rad) without heating and electrophoresis performed with Tris-Glycine-SDS (0.1% SDS) running buffer (BioRad). For native gels, Aβ samples were mixed 1:2 with Native Sample Buffer (BioRad) without heating and electrophoresis performed with Tris-Glycine buffer (BioRad). After electrophoresis, transfer to Westran S PVDF membranes (Whatman) was performed using a MiniProtean II (BioRad) or iBlot system (Invitrogen).

For PICUP experiments, immunoblotting was performed using near infrared fluorescence imaging as follows. The membrane was blocked with a Near Infrared Fluorescent Blocking Buffer (Rockland Immunocytochemicals, Gilbertsville, PA) for 1 h at room temperature, incubated with mAb 6E10 (1:5000 dilution) overnight at 4 °C, and in Mouse IgG IRDye800 Conjugated Rabbit Polyclonal solution (Rockland Immunocytochemicals; 1:15,000 dilution) for 1 h at room temperature. Infrared image was performed with the Odyssey Infrared Imaging System (LI-COR Biosciences, Lincoln, NE).

For non-PICUP Western Blots, immunoblotting with 6E10 and chemiluminescent imaging were performed as described in previous work [7]. Briefly, this process involved 1 h of blocking in 10% non-fat dry milk in phosphate buffered saline, overnight incubation with a 1:5000 dilution of 1 mg/ml 6E10 primary antibody (Covance) at 4 °C, and a one hour incubation with a 1:10,000 dilution of horseradish peroxidase conjugated goat anti-mouse IgG1 secondary antibody (Vector Labs). Membranes were developed in SuperSignal West Pico chemiluminescent substrate and bands detected on CL-XPosure film (Thermo Scientific). Immunoblotting for SDS-PAGE shown in the present work was performed using the visible-fluorescence method of the goat-antimouse Western Dot 625 Kit (Invitrogen W10132). The procedure followed the instructions in the kit and used a 1:1000 dilution of 1 mg/ml 6E10 primary antibody and 1:1000 dilution of goat anti-mouse IgG1 secondary antibody. Imaging was performed with a Gel Logic 100 Digital Imaging System (Kodak) using a Dark Reader Transilluminator (Clare Chemical).

### 4.5 Aggregation assays

Conversion of soluble Aβ species into insoluble aggregates was assessed in the Aβ incubation through measurement of Fraction Soluble, calculated using Eq. (1) [3]

(1) $$\mathrm{Fraction}\;\text{Soluble}=\frac{A_{280}^{t=n}}{A_{280}^{t=0}}$$

In Eq. (1), $A_{280}^{t=0}$ is the 280 nm absorbance on Day 0 and $A_{280}^{t=n}$ is the 280 nm absorbance on Day “*n*”, measured after centrifugation at 12,000×*g*. The experiment was run in quadruplet (*n*=4).

During the time course of the OAβ incubation, Thioflavin T (ThT) binding was determined in the raw preparation and also in a centrifuged portion of the preparation. Although not entirely specific, ThT binds amyloid fibrils quantitatively and produces an increase in fluorescence yield proportional to the extent of peptides in the amyloid state [3,8,9]. While ThT binding does not provide a direct measurement of amyloid fibril concentration, it is an accurate gauge of the fraction of amyloid fibrils formed in a preparation solution at a given time versus that present earlier or later. In the present study, a 40 μl aliquot of each Aβ sample was added to 1 ml of 25 μM ThT (100 mM sodium phosphate, 100 mM NaCl, pH 7.4) and incubated for 30 min [3]. ThT binding was quantified using Eq. (2)

(2) $$\mathrm{ThT}\;\;\text{Binding}=\frac{\begin{array}{ccc} I_{T+S} & - & I_{B+S} \end{array}}{\begin{array}{ccc} I_{T+B} & - & I_{B} \end{array}}-1$$

In Eq. (2), *I*_T+S_ is the fluorescence (excitation 450 nm, emission 480 nm) of ThT with Aβ sample added, *I*_B+S_ is a fluorescence correction for residual light scattering consisting of 40 μl Aβ sample in 1 mL buffer, *I*_T+B_ is the intrinsic fluorescence of the 1 ml ThT solution with 40 μL of buffer added, and I_B_ is the buffer background. The experiment was run in quadruplet (*n*=4).

### 4.6 Electron microscopy

A 70 μL sample aliquot was incubated on a formar grid for 20 min, fixed with 1 added drop of 1% glutaraldehyde in 100 mM phosphate buffer and 10 min incubation, rinsed in distilled water, stained with 1% uranyl acetate for 10 min, rinsed again and dried. Approximately 10 scans within the field were collected for each sample.

### 4.7 Circular dichroism

CD spectra were acquired for 0.5 s every 1 nm over a wavelength range of 195–260 nm with sample in a quartz cuvette with a 0.1 cm pathlength. The CD spectra presented are the average of five such spectra collected in series.

### 4.8 Fluorescence resonance energy transfer (FRET) studies

FRET analysis of peptide conformation in soluble oligomers followed a published methodology [3]. For FRET studies, two samples were prepared and incubated as described in *Monomeric and Oligomeric Aβ Preparation* with 48 μM unlabeled Aβ plus 2 μM of one of two fluorescent-labeled Aβ peptides: (1) D-Aβ or (2) D-Aβ-A. In these peptides, D indicates a substitution of (o-aminobenzoyl)lysine for methionine at position 35 (fluorescence energy donor, EX_max_=320 nm, EM_max_=420 nm) and *A* indicates a substitution of o-nitrotyrosine for tyrosine at position 10 (nonfluorescent FRET acceptor).

On each day of analysis (Day 0, 21, 42, and 365), two aliquots from both D-Aβ and D-Aβ-A preparations were removed. One aliquot was measured directly and the second was micro-centrifuged at 12,000 rpm prior to analysis to remove insoluble aggregates. To track reaction progress and confirm consistency with the unlabeled Aβ oligomer incubation, both aliquots were analyzed with steady-state fluorescence, florescence anisotropy, light scattering, turbidity, and thioflavin T binding (not shown).

The primary determination of FRET efficiency was performed using the time resolved fluorescence decay lifetimes of D-Aβ and D-Aβ-A samples. After deconvolution with the instrument response function using Savuka software, the time-dependent fluorescence decay A(t) of soluble D-Aβ and D-Aβ-A peptides was well characterized using the single exponential fitting model shown in Eq. (3) [10]

(3) $$A(t)=A_{x}e^{-\frac{t}{\tau_{x}}}+A(\infty )$$

Eq. (3) shows the fitted fluorescence lifetime *τ*_x_, signal amplitude *A*_x_, and final signal value *A*(∞) of the fluorescence decay corresponding to either X=D-Aβ or X=D-Aβ-A [10]. The time resolved fluorescence decay lifetimes *τ*_D-Aβ_ and *τ*_D-Aβ-A_ were used to determine the FRET efficiency (*E*) as shown in Eq. (4) [11,12]

(4) $$E=1-\frac{\tau_{D-A\beta -A}}{\tau_{D-A\beta }}$$

Between values of 0.1–0.9, *E* is roughly proportional to the average D-A distance in the sample. The 1:24 ratio of labeled D-Aβ-A versus unlabeled Aβ used here was shown to suppress all intermolecular FRET between neighboring DA peptides in a prior study of polyglutamic acid [3]. Thus, *E* here provides a probe of Aβ molecular conformation reflected in the intramolecular D-A distance of D-Aβ-A.
